# Stroke genetics: prospects for personalized medicine

**DOI:** 10.1186/1741-7015-10-113

**Published:** 2012-09-27

**Authors:** Hugh S Markus

**Affiliations:** 1 Stroke and Dementia Research Centre, St. Georges University of London, London, UK

## Abstract

Epidemiologic evidence supports a genetic predisposition to stroke. Recent advances, primarily using the genome-wide association study approach, are transforming what we know about the genetics of multifactorial stroke, and are identifying novel stroke genes. The current findings are consistent with different stroke subtypes having different genetic architecture. These discoveries may identify novel pathways involved in stroke pathogenesis, and suggest new treatment approaches. However, the already identified genetic variants explain only a small proportion of overall stroke risk, and therefore are not currently useful in predicting risk for the individual patient. Such risk prediction may become a reality as identification of a greater number of stroke risk variants that explain the majority of genetic risk proceeds, and perhaps when information on rare variants, identified by whole-genome sequencing, is also incorporated into risk algorithms. Pharmacogenomics may offer the potential for earlier implementation of 'personalized genetic' medicine. Genetic variants affecting clopidogrel and warfarin metabolism may identify non-responders and reduce side-effects, but these approaches have not yet been widely adopted in clinical practice.

## Review

### Stroke: the scale of the problem

Stroke represents a major health problem. Every year in the USA, 795,000 people experience a new or recurrent stroke [1]. Mortality data from 2008 indicate that stroke accounted for 1 in 18 deaths in the USA [1]. The Framingham Study showed that 1 in 5 women and 1 in 6 men aged 55 to 75 years will experience stroke sometime during their life [2]. Although the incidence may be reducing in developed countries, it has been estimated that stroke mortality will double worldwide by 2020, owing to an ageing population and an increasing incidence in developing countries.

Stroke is not only one of the major causes of death, but is also the leading cause of long-term disability, making it very costly to the economy. It has been estimated that the cost of the 152,000 incident strokes annually in the UK, and the cost of looking after patients who have had previous stroke, is around £8 billion in total, including at least £3 billion in direct healthcare costs [3].

Cerebrovascular disease (CVD) also causes vascular dementia, which is not only an important cause of dementia in its own right, but also seems to act synergistically with Alzheimer's disease pathology, increasing the chance of resulting clinical dementia [4]. In addition, CVD is the most common cause of adult-onset epilepsy, and there is increasing evidence that vascular changes contribute to late-onset depression [5].

Conventional cardiovascular risk factors are important in stroke risk, and include hypertension, smoking, diabetes mellitus, hyperlipidaemia, and coexistent cardiovascular disease including ischemic heart disease and atrial fibrillation. However, conventional risk factors fail to account for all stroke risk, as the proportion of unexplained risk has been estimated at about 50%, although such estimates vary [6].

### Stroke as a syndrome not a single disease

Stroke describes the clinical syndrome of focal neurological loss of function, usually of sudden onset, resulting from disturbance in the blood supply to the brain. It can be caused by occlusion of, or hemorrhage from, a cerebral blood vessel. About 80% to 85% of stroke cases are ischemic, whereas 15% to 20% are hemorrhagic.

Cerebral hemorrhage can be caused by multiple pathologies. Most cerebral hemorrhages are primary intracerebral hemorrhages, and many of these are subcortical hemorrhages associated with hypertension [7]. However, many other pathologies can also cause intracerebral hemorrhage, including cerebral amyloid angiopathy and underlying structural lesions. A minority of cerebral hemorrhage cases results from subarachnoid hemorrhage, which is often associated with rupture of an intracerebral aneurysm.

Not only can cerebral hemorrhage be caused by multiple different pathologies, but ischemic stroke is also heterogeneous. The three main causes of ischemic stroke are large-artery stenosis, small-vessel disease (SVD), and cardioembolism [8].

• Large-artery stroke results from atherosclerotic plaque in the carotid, vertebral, or major intracerebral arteries. Plaques, often associated with stenosis, become unstable, resulting in formation of thrombus, which subsequently embolizes distally to occlude cerebral vessels.

• Cardioembolic stroke results from a variety of intracardiac pathologies including atrial fibrillation, cardiomyopathy, and thrombus at the site of previous myocardial infarction.

• SVD (lacunar stroke) affects the small perforating arteries supplying subcortical structures, and results in small lacunar infarcts affecting the white matter and deep grey matter nuclei. The major risk factor for SVD is hypertension, and the underlying pathologies described include both diffuse small-vessel arteriopathy (lipohyalinosis) and focal atheroma.

In addition to these three most common types of ischemic stroke, there are many other rarer causes, including carotid and vertebral dissection, vasculitis, and single-gene disorders [8].

Identifying individual stroke subtypes requires detailed investigation including brain imaging, imaging of the extracerebral and intracerebral vessels, and cardiac imaging. Despite this, in as many as 25% to 40% of patients with ischemic stroke, an underlying pathology cannot be found [9].

This heterogeneity of stroke implies that different pathological mechanisms and risk factors are responsible for different stroke subtypes. Recent genetic studies from stroke are consistent with this, as described later.

### The role of genetics in stroke risk

A number of single-gene disorders can result in both ischemic and hemorrhagic stroke, and these tend to cause specific stroke subtypes. The most common is cerebral autosomal dominant arteriopathy with subcortical infarcts and leukoencephalopathy (CADASIL), a monogenic cause of cerebral SVD. Although important to an individual patient, these monogenic causes of stroke are rare, and contribute little to overall population risk of stroke. [10]

Epidemiological studies suggest that genetic risk factors are important for common 'sporadic' stroke. The most robust data on the heritability of a disease come from twin studies, which have compared the incidence of stroke in monozygotic compared with dizygotic twins. Twin studies in stroke support a genetic predisposition, but the number of stroke cases in prospective twin studies is small and therefore, the confidence intervals are wide [11]. There is much more information from family-history studies, which show that a family history of stroke is more common in stroke cases than in stroke-free controls [11,12]. Such an association could be caused by recall bias, with stroke cases more likely to have identified a family history of stroke, but prospective data from the Framingham Study, where information on family history was taken before the onset of stroke, confirms this association [13]. However, an association with family history could also be caused by shared early life environment, and separating this from genetic risk is difficult. Family-history data suggest that the genetic risk may vary by stroke subtype, with stronger associations being reported for the large-artery disease and SVD subtypes [12,14]

### How can we identify genes for stroke?

Three main methods have been used; linkage, the candidate-gene approach, and genome-wide association studies (GWAS). Linkage relies on identifying associations between chromosomal markers and disease phenotype within families. Linkage is good at identifying genes that are associated with greatly increased risk, but is less successful in more common polygenic diseases, in which multiple genes each contribute a small amount to overall risk. Linkage techniques have identified many disease-causing genes, but these have been primarily single-gene disorders. Using linkage, a number of genes causing monogenic stroke, such as the *notch3 *gene causing CADASIL, have been discovered [15], but the technique has also been used to look for variants contributing to polygenic stroke. This approach found that variants in the phosphodiesterase 4D gene *PED4D *were associated with ischemic stroke in an Icelandic population, [16] but this could not be replicated in other European populations, suggesting that it is either not important in stroke as a whole, or is only important in specific populations. [17]

Until recently, the main technique used to look for genes predisposing to common stroke was the candidate-gene method. Using this method, genetic variants, usually single-nucleotide polymorphisms (SNPs), are identified in a 'candidate' gene that is thought to be involved in stroke risk. The frequency of the SNP is then compared between stroke patients and controls, using a case-control approach. Many candidate-gene studies on stroke have been published. but the results have been largely disappointing, with few associations replicated. This picture is common to the genetics of many other complex diseases. The reasons for this lack of success have been explored both in general, and specifically for stroke [18]. Important factors are likely to include small sample sizes; a failure to replicate positive associations, coupled with publication bias resulting in preferential publication of positive associations; and a failure to phenotype cases accurately. A further problem with candidate-gene studies is that associations can only be identified in genes already known and implicated in stroke risk; completely novel genes cannot be identified.

The field of complex genetics has been revolutionized by the GWAS approach, which uses microarray technology to genotype up to one million or more SNPs, spanning the whole genome, in an individual subject [19]. A case-control or cohort approach is then used to compare the frequency of individual SNPs between disease cases and controls, and this is combined with rigorous statistical multiple-comparison methods to account for the many associations tested. Unlike the candidate-gene method, GWAS allows associations between novel chromosomal loci and disease to be identified. This technology has been combined with a realization of the importance of both very large sample sizes and the need to replicate positive associations prior to publication. GWAS had resulted in more than 1,600 novel associations with many complex diseases being identified by September 2011 [20]. Novel genetic associations have been reported in many CVDs including myocardial infarction, hypertension, hyperlipidemia, and diabetes. Most genetic variants discovered using GWAS account for only a small increase in disease risk, with odds ratios (Ors) most often between 1.1 and 1.3. This means that large sample sizes are required to identify such variants, and has resulted in the formation of disease consortiums that combine data from multiple studies in meta-analyses. Some of these now have 50,000 or more samples, allowing identification of variants with progressively smaller ORs.

### Genome-wide association studies in stroke

GWAS in stroke have lagged behind those in other CVDs, perhaps because the heterogeneity of the stroke phenotype was thought to make the chance of success less likely; however, the approach is now identifying novel genetic variants for stroke. In this brief review, I will focus on ischemic stroke, although advances are also being made in cerebral hemorrhage and in the genetics of intracranial aneurysms.

Initial studies attempted to replicate GWAS associations that had been initially found in other diseases associated with increased stroke risk. Two variants (*PITX2 *and *ZFHX3*), which were initially associated with atrial fibrillation, have both been shown to be independent risk factors for ischemic stroke; associations are only apparent with cardioembolic stroke and not with other stroke subtypes [21,22]. A variant at chromosome 9p21, which was originally associated with myocardial infarction and coronary artery disease [23], was found to be associated with ischemic stroke across multiple populations, but this association was present only with large artery stroke [24].The same locus has been associated with aortic and intracranial aneurysms [25].

There have been fewer novel GWAS associations initially identified in stroke itself, although a number of large GWAS in ischemic stroke are now taking place. Recently, the Wellcome Trust Case Control Consortium 2 ischemic stroke GWAS identified a novel association at 7p21; the most likely underlying gene is *HDAC9*, encoding histone deacetylase 9 [26].This association was confined to the large artery stroke subtype. *HDAC9 *is a member of a large family of genes that encode proteins responsible for deacetylation of histones, and therefore regulate chromatin structure and gene transcription. The mechanism by which variants in *HDAC9 *increase large artery stroke risk is not immediately clear, although the specific association with large artery stroke might suggest that they act through increasing atherogenesis. Sodium valproate, which has HDAC inhibitory properties, has been shown to inhibit atheroclerosis in animal models [27], and intriguingly, sodium valproate therapy in humans has been associated with lower stroke and myocardial infarction rates compared with other anti-epileptics [28].

A GWAS on Japanese cases with ischemic stroke identified an SNP in a member of the protein kinase (PKC) family, PRKCH, which was associated with small-vessel stroke [29]. This was replicated in an independent cohort, and the association has been further replicated in a Chinese population [30], and also with MRI-determined silent brain infarction [31]. PRKCH is a serine/threonine kinase that regulates a variety of cellular functions including differentiation, proliferation, and apoptosis [29]. The SNP identified is very rare in white populations, and whether it contributes to disease risk in populations other than Japanese and Chinese, or whether the association can be replicated more widely, remains to be determined.

Most studies to date have used a case-control design. This design could be open to bias if the gene in question is associated with early mortality in a disease such as stroke, where there is a significant early mortality, and if samples are collected only from survivors. Cohort studies, in which subjects are followed for many years and those developing stroke are compared with those remaining stroke-free, avoid this bias; however such studies tend to have smaller numbers of stroke, and also subtyping can be difficult as the strokes may occur in multiple hospitals. A meta-analysis of prospective cohort GWAS studies reported an association with the 12p13 region [32], but this could not be replicated in a much larger case-control study [33] or in a second Swedish study [34]. This could be because the variant is associated with stroke mortality, but it could also represent a false-positive association.

An alternative design is to genotype more than one family member and use family-based analysis methods. These can involve parent and offspring trios or more extensive family structures, or be based on sibling pairs. The former are difficult to collect for a late-onset disease such as stroke; affected sibling-pair collections are more realistic, but even collecting these in stroke is challenging, and obtaining the large sample sizes required for GWAS has proved difficult [35].

In summary, GWAS studies have identified a few robust associations with ischemic stroke. The associations found to date have been for specific stroke subtypes, emphasizing the importance of careful phenotyping, and suggesting that different stroke subtypes have different pathophysiologic mechanisms and genetic risk-factor profiles.

The largest GWAS studies in stroke to date have comprised about 3,000 cases. Larger studies are underway. The Stroke Genetics Network (SiGN) study, funded by the National Institute of Neurological Disorders and Stroke, aims to genotype at least a further 6,000 cases. The Metastroke collaboration has brought together groups with GWAS data on ischemic stroke from throughout the world, and currently comprises about 14,000 cases and 50,000 controls.

### Future advances

Meta-analysis of GWAS data from tens of thousands of patients with stroke is likely to identify further variants, as it has for other complex diseases. If variants do indeed predispose to specific stroke subtypes, then even larger sample sizes may be required. For example, even though a sample size of 10,000 sounds large, it will only include approximately 2,000 individuals with large artery stroke.

The GWAS approach is suited to identify common variants, each of which contribute a small amount to disease risk. It is less effective at detecting rare variants, which might still be important in disease risk. Whole-genome sequencing enables these rare variants to be identified, and the cost of this technique is rapidly falling [36]. As yet, results are not available from this approach for stroke, although studies are underway. Many current sequencing studies limit coverage to sequencing of the exome, or protein coding part, of the genome. Exome sequencing has been successful in many rare, primarily monogenic, diseases and may offer a cost-effective way to screen for multiple single-gene causes of stroke in one assay. Studies are underway using exome sequencing to try to identify rare variants that may contribute to more common polygenic diseases [37]. Such studies may particularly benefit from the use of family-based approaches, to help separate causal from non-causal variants.

Another emerging area is epigenetics, although to date there have been few studies for stroke. Epigenetics describes the study of heritable changes in gene expression or cellular phenotype, which are caused by mechanisms other than changes in the underlying DNA sequence [38]. It therefore refers to functionally relevant modifications in the genome that do not involve the changed nucleotide sequence. Examples of such changes are DNA methylation and histone modification, both of which serve to regulate gene expression without altering the DNA structure. Methods are now becoming available to assess epigenetic changes. For example, array-based methods can be use for typing DNA methylation in large populations, and case-control studies similar to those being performed for GWAS are beginning to reveal interesting results [39].

### How can identifying genes for stroke help patients?

Even though monogenic stroke is rare, identifying the underlying gene can be important for the individual patient. In such diseases, a mutation in a specific gene results in disease, and most individuals with the mutation are likely to develop stroke or other clinical presentations of the disease at some stage in their life. Identifying the underlying mutation allows diagnosis, information on prognosis, and in some cases, specific treatments. It also enables counseling of other family members, and prenatal testing if desired. However, many monogenic forms of stroke are untreatable, and therefore, specialized genetic counseling is important before mutation testing. This is particularly important in asymptomatic individuals, or those with mild disease; for example, potential CADASIL patients who have migraine but have not yet developed stroke or dementia.

However, the vast majority of stroke is 'polygenic', with many genes thought to be involved, each conferring a small risk and probably interacting with multiple environmental risk factors to cause disease. How can such genetic knowledge benefit patients? Can we really offer personalized medicine in which the genetic profile in an individual provides useful information on stroke risk? Genetic testing for polygenic diseases is already being developed, and indeed, some gene tests for cardiovascular disease have already been marketed, with the individual purchasing a test online and sending off a saliva swab for DNA extraction and testing for a number of at-risk SNPs. However, the clinical use of such tests has been questioned. Genetic epidemiological studies have suggested that the sibling relative risk for stroke is approximately 2 to 3, with the higher estimate applying if younger patients are considered [40]. Assuming that the genetic variants identified confer ORs of between 1.1 and 1.2, it has been calculated that 100 to 300 different genetic variants explain this degree of risk [41]. Therefore, panels that include only 5 to 10 or so variants will explain only a small proportion of overall disease risk and so be poor disease predictors. The genetic variants for stroke described to date account for only a small proportion of overall stroke risk. Therefore, even when combined, their predictive value is low, and even if an individual has these variants, they may not develop stroke during their lifetime, whereas people without these variants could be at risk of stroke. Until we have a more complete understanding of the molecular basis of genetic variation, such predictive testing is likely to provide limited information.

There are also questions about the usefulness of such personalized testing in patients with complex diseases such as stroke. We already know many risk factors for stroke, such as hypertension and smoking, but despite their importance, patient compliance is often suboptimal. Unless there are specific novel treatments for individual genetic variants, it is likely that the advice given to a patient identified as having a high genetic risk of stroke would merely be to adhere more closely to cardiovascular risk-factor prevention, yet it is unclear whether such high-risk patients would indeed do so. Furthermore, there is the possibility that patients deemed to have low genetic risk might pay less attention to general risk-factor prevention and therefore, expose themselves to increased risk. There has also been concern over the psychological consequences of testing.

Therefore, the clinical use of genetic profiling of stroke risk is likely to be some way in the future. In the more immediate future, identifying novel genetic variants may contribute to treating disease by identifying new pathways involved in the pathogenesis of stroke. Using information gained from GWAS to develop novel treatments for complex diseases is beginning to bear fruit in other diseases such as macular degeneration and Crohn's disease [42,43]. One criticism is that the genetic associations identified are unlikely to be important in view of the small increase in risk (OR) associated with each one. However, it is relevant that the total variance in disease risk explained by genes involved in pathological processes targeted by already established successful currently available drugs, such as statins for hypercholesterolaemia, sulfonylureas for diabetes, and estrogens for bone density, is often small [44].

### Pharmacogenomics

One area where personalized genetic medicine may have earlier application is pharmacogenomics. Genetic variation influences drug metabolism, and thus both drug efficacy and risk of drug-related adverse effects (AEs). Pharmacogenomics uses an individual's genotype to assist in choosing therapies and identifying the optimal dose, with the aim of ensuring maximum efficacy with minimal AEs [45]. In addition, it can provide new insights into the mechanisms of drug action, and therefore contribute to the development of new therapeutic agents. As yet, pharmacogenomics has had little effect on routine clinical stroke care in most countries, but two potential applications in tailoring anti-platelet therapy and warfarin dosage in patients with CVD, including stroke, have been proposed, for the drugs clopidogrel and warfarin.

### Clopidogrel

Clopidogrel is widely used for prevention of secondary stroke. It is more effective than aspirin alone [46], and has similarly effectiveness to the combination of aspirin and dypridamole in long-term secondary prevention [47]. Approximately 5 to 30% of clopidogrel-treated patients exhibit low or no reactivity to clopidogrel, which is referred to as **'**clopidogrel resistance.**' **Clopidogrel requires transformation into an active metabolite by cytochrome P450 (CYP) for its anti-platelet effect. Different CYP isoenzymes are responsible for clopidogrel activation, and among these, CYP2C19 has been found to play a key role. Carriers of at least one CYP2C19*2 reduced-function allele (about 25 to 30% of the population) have a one-third reduction in the active metabolite of clopidogrel compared with non-carriers, whereas the 2% of individuals homozygous for the polymorphism have a much greater reduction [48]. The variant has been associated with a corresponding reduction in platelet inhibition [48].

A number of reports have suggested that this variant was associated with increased cardiovascular events in patients on clopidogrel, particularly after coronary stenting, where there is a high risk of stent thrombolysis. This led to the US Food and Drug Administration (FDA) announcing in 2010 that clopidogrel would receive a boxed warning in the prescribing information, which cautioned that slow metabolism of clopidogrel was associated with higher cardiovascular event rates, and suggested that genetic testing could identify individuals who were slow metabolizers, thereby allowing physicians to implement 'alternative treatment strategies.' [49]. This FDA announcement was controversial. The American Heart Association (AHA) and American College of Cardiology (ACC) issued a consensus statement that concluded 'The evidence base is insufficient to recommend either routine genetic or platelet function testing at the present time' [50]. This controversy has continued, with arguments being made both for [51] and against [52] testing, although these have been primarily applied to treatment in stented individuals and not in non-stented patients with coronary ischaemia or patients with stroke.

A recent systematic review and meta-analysis identified 32 studies of 42,016 patients reporting 3,545 cardiovascular events and 1,413 bleeding events [53]. Six studies were randomized trials with comparison against another treatment (effect-modification design) whereas the remaining 26 studies comprised individuals exposed to clopidogrel with no control arm (treatment-only design). In the analysis of treatment-only studies, individuals with one or more *CYP2C19 *alleles had a lower risk of bleeding, and a higher risk of cardiovascular events. However, when analyses were restricted to studies with 200 or more events, the association was no longer significant, consistent with publication bias. In studies that included a control arm, the *CYP2C19 *genotype was not associated with modification of the effect of clopidogrel on cardiovascular end points or bleeding.

Even if an association can be shown between a genetic variant and drug efficacy, the most powerful evidence for the use of such a genetic test in clinical practice is a randomized, controlled trial that compare a strategy of modifying treatment, based on the results of genetic screening, with standard care (that is, no testing). Such trials will not only provide reliable estimates of the effect of genotype on drug response but will also take into account the potential effect of the testing procedure itself on patient outcomes [52].

Most studies of the *CYP2C19 *polymorphisms have been in coronary artery disease, but in a genetic substudy of the Clopidogrel for High Atherothrombotic Risk and Ischemic Stabilization, Management, and Avoidance (CHARISMA) study, about 20% of the 4,819 genotyped patients had ischemic stroke at entry [54]. Carriers of *CYP2C19 *loss-of-function alleles did not have an increased rate of ischemic events, but did have a significantly lower rate of any bleeding when on clopidogrel.

The story of a personalized pharmacogenomic approach to clopidogrel therapy illustrates the difficulties in implementing such an approach, and the need for rigorous assessment of its benefit and effect on clinical outcome. This does not mean that the approach may not prove useful in the longer term. The *CYP2C19 *loss-of-function alleles account for only 12% of the variability in response to clopidogrel, whereas 72% of the variability is heritable [52]. Genetic testing of a wider range of variants that better captures this heritability is likely to provide more predictive information.

### Warfarin

Warfarin reduces the risk of stroke in patients with non-valvular atrial fibrillation, and is also used in patients with other cardiac lesions associated with a high risk of cardioembolism, including prosthetic heart valves and mural thrombus. The high variability in drug response means that blood monitoring of coagulation with the international normalized ratio (INR) is required, but there is a narrow therapeutic index, and there is a a risk of thrombosis with under-anti-coagulation and of hemorrhage with over-anti-coagulation. Warfarin is the second leading drug-related reason for emergency department visits [55], and the most frequently cited reason for drug-related mortality [56]. Therefore, methods to improve the safety and effectiveness of warfarin therapy would have wide application.

A number of genetic variants have to shown to influence warfarin levels [45]. Warfarin is a racemic mixture, with S-warfarin being more potent than R-warfarin. CYP2C9 is a hepatic drug-metabolizing enzyme in the CYP450 superfamily, and is the primary metabolizing enzyme of S-warfarin. Two common CYP2C9 allozymes have markedly reduced enzyme activity. It was shown that patients who required a low final dose of warfarin on the basis of INR values often carried one or two of these two common *CYP2C9 *variant alleles, and were at increased risk for hemorrhage during warfarin therapy, presumably because they metabolize the drug more slowly [45]. Vitamin K epoxide reductase complex subunit 1, is the target for warfarin-based anticoagulants, and SNPs in the *VKORC1 *gene are also associated with the dose of warfarin required to achieve a target INR value. Together, the *CYP2C9 *and *VKORC1 *polymorphisms explain about 30 to 40% of the total variation in the final warfarin dose [57].

To assess the added contribution of testing for these genetic variants, the clinical and genetic data from 4,043 patients were used to create a dose algorithm that was based on clinical variables only, and an algorithm in which genetic information was added to the clinical variables [58]. This was validated in a second cohort of 1,009 subjects. Use of the pharmacogenetic algorithm produced dose recommendations that were significantly closer to the required stable therapeutic dose than those derived from the clinical algorithm, particularly for patients who required unusually high or low warfarin doses.

Supporting this approach, a mulitcenter national study prospectively collected data on rate of hospitalization over a 6-month period in 896 patients receiving warfarin genotyping, and compared this with 2,688 matched historical controls [59]. The genotyped cohort had 31% fewer hospitalizations overall, and 28% fewer hospitalizations for bleeding or thromboembolism.

In February 2010, the FDA revised the label on warfarin, providing genotype-specific ranges of doses, and suggesting that genotypes be taken into consideration when the drug is prescribed. *CYP2C9 *and *VKORC1 *genotyping is now clinically available. as are online and web-based algorithms incorporating genotypic information to calculate dosage [60]. Despite this, implementation of this genetic testing in clinical practice has been slow. It has been argued that a truly randomized trial is required to confirm the effect on clinical management and to examine cost-effectiveness. In addition, new anticoagulants with a wider therapeutic range and acting by different mechanisms have been shown to be as effective as warfarin in stroke prevention, and may be preferred for patients for whom warfarin therapy is difficult or has anticipated side-effects.

### Promise of pharmacogenomics

The cases of clopidogrel and warfarin demonstrate the promise of pharmacogenomics, but also the difficulties in evaluating the clinical effect of such an approach. With the increasing reliance on evidence-based medicine and large randomized trials, it is likely that a similar degree of evidence will be required before such approaches are widely implemented. Nevertheless, this is an area that could significantly improve targeting of therapies and reduce side-effects.

## Conclusions

Epidemiologic evidence supports genetic factors being important in stroke risk. Recent advances, primarily GWAS, are transforming what we know about the genetics of multifactorial stroke, and are identifying novel stroke genes. The findings to date suggest that different stroke subtypes have different genetic architecture. Novel genes associated with stroke may identify novel pathways involved in stroke pathogenesis, and suggest new treatment approaches. The already identified genetic variants explain only a small proportion of overall stroke risk, and therefore are not currently useful in predicting risk in the individual patient. Predicting stroke risk in the individual patient may become a possibility if a greater number of stroke risk variants that explain the majority of genetic risk can be identified, and if information on rare variants, identified by whole-genome sequencing, is also incorporated into risk algorithms. Pharmacogenomics may offer the potential for earlier implementation of 'personalized genetic' medicine. Genetic variants affecting clopidogrel and warfarin metabolism may identify non-responders and reduce side-effects, but their use has not yet been widely adopted in clinical practice.

## Competing interests

The author declares that they have no competing interests.

## Author's information

HM is Professor of Neurology at St George's University of London and an Honorary Consultant Neurologist/Stroke Physician at St George's Hospital. He spends half his time looking after patients with stroke, and the other half on academic activities. His research interests involve applying genetic and imaging techniques to investigate the pathogenesis of stroke, and to implement new stroke therapies in clinical trials.

## Note

Table of contents

Abstract

Keywords

Stroke - the scale of the problem

Stroke as a syndrome not a single disease

The role of genetics in stroke risk

How can we Identify genes for stroke?

GWAS in stroke

Future advances

How can identifying genes for stroke help patients?

Pharmacogenomics

Conclusions

Competing interests

Acknowledgements

References

## Pre-publication history

The pre-publication history for this paper can be accessed here:

http://www.biomedcentral.com/1741-7015/10/113/prepub

## References

[B1] Roger VL Go AS Lloyd-Jones DM Benjamin EJ Berry JD Borden WB Bravata DM Dai S Ford ES Fox CS Fullerton HJ Gillespie C Hailpern SM Heit JA Howard VJ Kissela BM Kittner SJ Lackland DT Lichtman JH Lisabeth LD Makuc DM Marcus GM Marelli A Matchar DB Moy CS Mozaffarian D Mussolino ME Nichol G Paynter NP Soliman EZ Sorlie PD Sotoodehnia N Turan TN Virani SS Wong ND Woo D Turner MB American Heart Association Statistics Committee and Stroke Statistics Subcommittee. Heart disease and stroke statistics--2012 update: a report from the American Heart Association Circulation 2012 125 e2 220 22179539 10.1161/CIR.0b013e31823ac046 PMC4440543

[B2] Seshadri S Beiser A Kelly-Hayes M Kase CS Au R Kannel WB Wolf PA The lifetime risk of stroke: estimates from the Framingham Study Stroke 2006 37 345 350 10.1161/01.STR.0000199613.38911.b2 16397184

[B3] Progress in improving stroke care National Audit Office 2010 Available online http://www.nao.org.uk/publications/0910/stroke.aspx

[B4] Viswanathan A Rocca WA Tzourio C Vascular risk factors and dementia: how to move forward? Neurology 2009 72 368 74 10.1212/01.wnl.0000341271.90478.8e 19171835 PMC2677504

[B5] O'Brien JT Firbank MJ Krishnan MS van Straaten EC van der Flier WM Petrovic K Pantoni L Simoni M Erkinjuntti T Wallin A Wahlund LO Inzitari D LADIS Group White matter hyperintensities rather than lacunar infarcts are associated with depressive symptoms in older people: the LADIS study Am J Geriatr Psychiatry 2006 14 834 41 10.1097/01.JGP.0000214558.63358.94 17001023

[B6] Sacco RL Ellenberg JH Mohr JP Tatemichi TK Hier DB Price TR Wolf PA Infarcts of undetermined cause: the NINCDS stroke data bank Ann Neurol 1989 25 382 90 10.1002/ana.410250410 2712533

[B7] Qureshi AI Mendelow AD Hanley DF Intracerebral haemorrhage Lancet 2009 373: 1632 44 19427958 10.1016/S0140-6736(09)60371-8 PMC3138486

[B8] Markus HS Markus HS An Introduction to stroke Stroke Genetics 2003 Oxford University Press, Oxford, UK 1 30

[B9] Lanfranconi S Markus HS Stroke subtyping for genetic association studies? A comparison of the CCS and TOAST classifications Int J Stroke 2012 in press 10.1111/j.1747-4949.2012.00780.x 22520626

[B10] Markus HS Unravelling the genetics of ischaemic stroke PLoS Med 2010 7 e1000225 10.1371/journal.pmed.1000225 20209141 PMC2830452

[B11] Jerrard-Dunne P Cloud G Hassan A Markus HS Evaluating the genetic component of ischemic stroke subtypes: a family history study Stroke 2003 34 1364 9 10.1161/01.STR.0000069723.17984.FD 12714707

[B12] Flossmann E Schulz UGR Rothwell PM A systematic review of the methods and results of studies of the genetic epidemiology of ischaemic stroke Stroke 2004 35 212 227 14684773 10.1161/01.STR.0000107187.84390.AA

[B13] Seshadri S Beiser A Pikula A Himali JJ Kelly-Hayes M Debette S DeStefano AL Romero JR Kase CS Wolf PA Parental occurrence of stroke and risk of stroke in their children: the Framingham study Circulation 2010 121 1304 1312 10.1161/CIRCULATIONAHA.109.854240 20212282 PMC2860311

[B14] Polychronopoulos P Gioldasis G Ellul J Metallinos IC Lekka NP Paschalis C Papapetropoulos T Family history of stroke in stroke types and subtypes J Neurol Sci 2002 195 117 122 10.1016/S0022-510X(01)00691-8 11897241

[B15] Joutel A Corpechot C Ducros A Vahedi K Chabriat H Mouton P Alamowitch S Domenga V Cécillion M Maréchal E Maciazek J Vayssière C Cruaud C Cabanis EA Ruchoux MM Weissenbach J Bach JF Bousser MG Tournier-Lasserve E Notch3 mutations in CADASIL, a hereditary adult-onset condition causing stroke and dementia Nature 1996 383 707 710 10.1038/383707a0 8878478

[B16] Gretarsdottir S Thorleifsson G Reynisdottir ST Manolescu A Jonsdottir S Jonsdottir T Gudmundsdottir T Bjarnadottir SM Einarsson OB Gudjonsdottir HM Hawkins M Gudmundsson G Gudmundsdottir H Andrason H Gudmundsdottir AS Sigurdardottir M Chou TT Nahmias J Goss S Sveinbjörnsdottir S Valdimarsson EM Jakobsson F Agnarsson U Gudnason V Thorgeirsson G Fingerle J Gurney M Gudbjartsson D Frigge ML Kong A Stefansson K Gulcher JR The gene encoding phosphodiesterase 4D confers risk of ischaemic stroke Nat Genet 2003 353 131 138 14517540 10.1038/ng1245

[B17] Bevan S Dichgans M Gschwendtner A Kuhlenbäumer G Ringelstein EB Markus HS Variation in the PDE4D Gene and Ischemic Stroke Risk. A Systematic Review and Meta-analysis on 5200 Cases and 6600 Controls Stroke 2008 39 1966 1971 10.1161/STROKEAHA.107.509992 18420948

[B18] Dichgans M Markus HS Genetic association studies in stroke: methodological issues and proposed standard criteria Stroke 2005 36 2027 2031 10.1161/01.STR.0000177498.21594.9e 16051898

[B19] Hardy J Singleton A Genomewide association studies and human disease N Engl J Med 2009 360 1759 68 10.1056/NEJMra0808700 19369657 PMC3422859

[B20] A Catalog of Published Genome-Wide Association Studies http://www.genome.gov/gwastudies/

[B21] Gretarsdottir S Thorleifsson G Manolescu A Styrkarsdottir U Helgadottir A Gschwendtner A Kostulas K Kuhlenbäumer G Bevan S Jonsdottir T Bjarnason H Saemundsdottir J Palsson S Arnar DO Holm H Thorgeirsson G Valdimarsson EM Sveinbjörnsdottir S Gieger C Berger K Wichmann HE Hillert J Markus H Gulcher JR Ringelstein EB Kong A Dichgans M Gudbjartsson DF Thorsteinsdottir U Stefansson K Risk variants for atrial fibrillation on chromosome 4q25 associate with ischemic stroke Ann Neurol 2008 64 402 409 10.1002/ana.21480 18991354

[B22] Gudbjartsson DF Holm H Gretarsdottir S Thorleifsson G Walters GB Thorgeirsson G Gulcher J Mathiesen EB Njølstad I Nyrnes A Wilsgaard T Hald EM Hveem K Stoltenberg C Kucera G Stubblefield T Carter S Roden D Ng MC Baum L So WY Wong KS Chan JC Gieger C Wichmann HE Gschwendtner A Dichgans M Kuhlenbäumer G Berger K Ringelstein EB Bevan S Markus HS Kostulas K Hillert J Sveinbjörnsdóttir S Valdimarsson EM Løchen ML Ma RC Darbar D Kong A Arnar DO Thorsteinsdottir U Stefansson K A sequence variant in ZFHX3 on 16q22 associates with atrial fibrillation and ischemic stroke Nat Genet 2009 41 876 878 10.1038/ng.417 19597491 PMC2740741

[B23] Helgadottir A Thorleifsson G Manolescu A Gretarsdottir S Blondal T Jonasdottir A Jonasdottir A Sigurdsson A Baker A Palsson A Masson G Gudbjartsson DF Magnusson KP Andersen K Levey AI Backman VM Matthiasdottir S Jonsdottir T Palsson S Einarsdottir H Gunnarsdottir S Gylfason A Vaccarino V Hooper WC Reilly MP Granger CB Austin H Rader DJ Shah SH Quyyumi AA Gulcher JR Thorgeirsson G Thorsteinsdottir U Kong A Stefansson K A common variant on chromosome 9p21 affects the risk of myocardial infarction Science 2007 316 1491 3 10.1126/science.1142842 17478679

[B24] Gschwendtner A Bevan S Cole JW Plourde A Matarin M Ross-Adams H Meitinger T Wichmann E Mitchell BD Furie K Slowik A Rich SS Syme PD MacLeod MJ Meschia JF Rosand J Kittner SJ Markus HS Müller-Myhsok B Dichgans M Sequence variants on chromosome 9p21.3 confer risk for atherosclerotic stroke Ann Neurol 2009 65 531 539 10.1002/ana.21590 19475673 PMC2702695

[B25] Helgadottir A Thorleifsson G Magnusson KP Grétarsdottir S Steinthorsdottir V Manolescu A Jones GT Rinkel GJ Blankensteijn JD Ronkainen A Jääskeläinen JE Kyo Y Lenk GM Sakalihasan N Kostulas K Gottsäter A Flex A Stefansson H Hansen T Andersen G Weinsheimer S Borch-Johnsen K Jorgensen T Shah SH Quyyumi AA Granger CB Reilly MP Austin H Levey AI Vaccarino V Palsdottir E Walters GB Jonsdottir T Snorradottir S Magnusdottir D Gudmundsson G Ferrell RE Sveinbjornsdottir S Hernesniemi J Niemelä M Limet R Andersen K Sigurdsson G Benediktsson R Verhoeven EL Teijink JA Grobbee DE Rader DJ Collier DA Pedersen O Pola R Hillert J Lindblad B Valdimarsson EM Magnadottir HB Wijmenga C Tromp G Baas AF Ruigrok YM van Rij AM Kuivaniemi H Powell JT Matthiasson SE Gulcher JR Thorgeirsson G Kong A Thorsteinsdottir U Stefansson K The same sequence variant on 9p21 associates with myocardial infarction, abdominal aortic aneurysm and intracranial aneurysm Nat Genet 2008 40 217 24 10.1038/ng.72 18176561

[B26] International Stroke Genetics Consortium (ISGC); Wellcome Trust Case Control Consortium 2 (WTCCC2) Bellenguez C Bevan S Gschwendtner A Spencer CC Burgess AI Pirinen M Jackson CA Traylor M Strange A Su Z Band G Syme PD Malik R Pera J Norrving B Lemmens R Freeman C Schanz R James T Poole D Murphy L Segal H Cortellini L Cheng YC Woo D Nalls MA Müller-Myhsok B Meisinger C Seedorf U Ross-Adams H Boonen S Wloch-Kopec D Valant V Slark J Furie K Delavaran H Langford C Deloukas P Edkins S Hunt S Gray E Dronov S Peltonen L Gretarsdottir S Thorleifsson G Thorsteinsdottir U Stefansson K Boncoraglio GB Parati EA Attia J Holliday E Levi C Franzosi MG Goel A Helgadottir A Blackwell JM Bramon E Brown MA Casas JP Corvin A Duncanson A Jankowski J Mathew CG Palmer CN Plomin R Rautanen A Sawcer SJ Trembath RC Viswanathan AC Wood NW Worrall BB Kittner SJ Mitchell BD Kissela B Meschia JF Thijs V Lindgren A Macleod MJ Slowik A Walters M Rosand J Sharma P Farrall M Sudlow CL Rothwell PM Dichgans M Donnelly P Markus HS Genome-wide association study identifies a variant in HDAC9 associated with large vessel ischemic stroke Nat Genet 2012 44 328 33 10.1038/ng.1081 22306652 PMC3303115

[B27] Bowes AJ Khan MI Shi Y Robertson L Werstuck GH Valproate attenuates accelerated atherosclerosis in hyperglycemic apoE-deficient mice: evidence in support of a role for endoplasmic reticulum stress and glycogen synthase kinase-3 in lesion development and hepatic steatosis Am J Pathol 2009 174 330 42 10.2353/ajpath.2009.080385 19095952 PMC2631345

[B28] Olesen JB Abildstrøm SZ Erdal J Gislason GH Weeke P Andersson C Torp-Pedersen C Hansen PR Effects of epilepsy and selected antiepileptic drugs on risk of myocardial infarction, stroke, and death in patients with or without previous stroke: a nationwide cohort study Pharmacoepidemiol Drug Saf 2011 20 964 71 21766386 10.1002/pds.2186

[B29] Kubo M Hata J Ninomiya T Matsuda K Yonemoto K Nakano T Matsushita T Yamazaki K Ohnishi Y Saito S Kitazono T Ibayashi S Sueishi K Iida M Nakamura Y Kiyohara Y A nonsynonymous SNP in PRKCH (protein kinase C eta) increases the risk of cerebral infarction Nat Genet 2007 39 212 7 10.1038/ng1945 17206144

[B30] Wu L Shen Y Liu X Ma X Xi B Mi J Lindpaintner K Tan X Wang X The 1425G/A SNP in PRKCH is associated with ischemic stroke and cerebral hemorrhage in a Chinese population Stroke 2009 40: 2973 6 19520989 10.1161/STROKEAHA.109.551747

[B31] Serizawa M Nabika T Ochiai Y Takahashi K Yamaguchi S Makaya M Kobayashi S Kato N Association between PRKCH gene polymorphisms and subcortical silent brain infarction Atherosclerosis 2008 199 340 5 10.1016/j.atherosclerosis.2007.11.009 18164711

[B32] Ikram MA Seshadri S Bis JC Fornage M DeStefano A Aulchenko YS Debette S Lumley T Folsom AR van den Herik EG Bos MJ Beiser A Cushman M Launer LJ Shahar E Struchalin M Du Y Glazer NL Rosamond WD Rivadeneira F Kelly-Hayes M Lopez OL Coresh J Hofman A DeCarli C Heckbert SR Koudstaal PJ Yang Q Smith NL Kase CS Rice K Haritunians T Roks G de Kort PLM Taylor KD de Lau LM Oostra BA Uitterlinden AG Rotter JI Boerwinkle E Psaty BM Mosley TH van Duijn CM Breteler MB Longstreth WT Jr Wolf PA Genome-wide Association Studies of Incident Total Stroke and Ischemic Stroke: Meta-analysis and Replication from the CHARGE Consortium New Engl J Med 2009 360 1718 1728 10.1056/NEJMoa0900094 19369658 PMC2768348

[B33] International Stroke Genetics Consortium; Wellcome Trust Case-Control Consortium 2: Failure to validate association between 12p13 variants and ischemic stroke N Engl J Med 2010 362 1547 50 20410525 10.1056/NEJMc0910050 PMC2978046

[B34] Olsson S Melander O Jood K Smith JG Lövkvist H Sjögren M Engström G Norrving B Lindgren A Jern C International Stroke Genetics Consortium (ISGC) Genetic variant on chromosome 12p13 does not show association to ischemic stroke in 3 Swedish case-control studies Stroke 2011 42 214 6 10.1161/STROKEAHA.110.594010 21148441

[B35] Meschia JF Nalls M Matarin M Brott TG Brown RD Jr Hardy J Kissela B Rich SS Singleton A Hernandez D Ferrucci L Pearce K Keller M Worrall BB Siblings With Ischemic Stroke Study Investigators. Siblings with ischemic stroke study: results of a genome-wide scan for stroke loci Stroke 2011 42 2726 32 10.1161/STROKEAHA.111.620484 21940970 PMC3251509

[B36] Ng PC Kirkness EF Whole genome sequencing Methods Mol Biol 2010 628 215 26 10.1007/978-1-60327-367-1_12 20238084

[B37] Singleton AB Exome sequencing: a transformative technology Lancet Neurol 2011 10 942 6 10.1016/S1474-4422(11)70196-X 21939903 PMC3302356

[B38] Shirodkar AV Marsden PA Epigenetics in cardiovascular disease Curr Opin Cardiol 2011 26 209 15 10.1097/HCO.0b013e328345986e 21415727 PMC5303626

[B39] Ku CS Naidoo N Wu M Soong R Studying the epigenome using next generation sequencing J Med Genet 2011 48 721 30 10.1136/jmedgenet-2011-100242 21825079

[B40] Hassan A Sham P Markus HS Planning genetic studies in human stroke: Sample size estimates based on family history data Neurology 2002 58 1483 88 10.1212/WNL.58.10.1483 12034783

[B41] Kraft P Hunter DJ Genetic risk prediction-are we there yet? N Engl J Med 2009 360 1701 3 10.1056/NEJMp0810107 19369656

[B42] Rohrer B Long Q Coughlin B Wilson RB Huang Y Qiao F Tang PH Kunchithapautham K Gilkeson GS Tomlinson S A targeted inhibitor of the alternative complement pathway reduces angiogenesis in a mouse model of age-related macular degeneration Invest Ophthalmol Vis Sci 2009 50 3056 64 10.1167/iovs.08-2222 19264882 PMC2908507

[B43] Klionsky DJ Crohn's disease, autophagy, and the Paneth cell N Engl J Med 2009 360 1785 6 10.1056/NEJMcibr0810347 19369659 PMC2832908

[B44] Hirschhorn JN Genomewide association studies - illuminating biological pathways New Eng J Med 2009 360 1699 1701 10.1056/NEJMp0808934 19369661

[B45] Wang L McLeod HL Weinshilboum RM Genomics and drug response N Engl J Med 2011 364 1144 53 10.1056/NEJMra1010600 21428770 PMC3184612

[B46] CAPRIE Steering Committee A randomised, blinded, trial of clopidogrel versus aspirin in patients at risk of ischaemic events (CAPRIE) Lancet 1996 348 1329 39 8918275 10.1016/s0140-6736(96)09457-3

[B47] Diener HC Sacco RL Yusuf S Cotton D Ounpuu S Lawton WA Palesch Y Martin RH Albers GW Bath P Bornstein N Chan BP Chen ST Cunha L Dahlöf B De Keyser J Donnan GA Estol C Gorelick P Gu V Hermansson K Hilbrich L Kaste M Lu C Machnig T Pais P Roberts R Skvortsova V Teal P Toni D VanderMaelen C Voigt T Weber M Yoon BW Prevention Regimen for Effectively Avoiding Second Strokes (PRoFESS) study group Effects of aspirin plus extended-release dipyridamole versus clopidogrel and telmisartan on disability and cognitive function after recurrent stroke in patients with ischaemic stroke in the Prevention Regimen for Effectively Avoiding Second Strokes (PRoFESS) trial: a double-blind, active and placebo-controlled study Lancet Neurol 2008 7 875 84 10.1016/S1474-4422(08)70198-4 18757238 PMC2772657

[B48] Mega JL Close SL Wiviott SD Shen L Hockett RD Brandt JT Walker JR Antman EM Macias W Braunwald E Sabatine MS Cytochrome p-450 polymorphisms and response to clopidogrel N Engl J Med 2009 360 354 362 10.1056/NEJMoa0809171 19106084

[B49] Nissen SE Pharmacogenomics and clopidogrel: irrational exuberance? JAMA 2011 306 2727 8 10.1001/jama.2011.1865 22203545

[B50] Holmes DR Jr Dehmer GJ Kaul S Leifer D O'Gara PT Stein CM ACCF/AHA clopidogrel clinical alert: approaches to the FDA "boxed warning": a report of the American College of Cardiology Foundation Task Force on clinical expert consensus documents and the American Heart Association endorsed by the Society for Cardiovascular Angiography and Interventions and the Society of Thoracic Surgeons J Am Coll Cardiol 2010 56 321 341 10.1016/j.jacc.2010.05.013 20633831

[B51] Sibbing D Bernlochner I Kastrati A Paré G Eikelboom JW Current evidence for genetic testing in clopidogrel-treated patients undergoing coronary stenting Circ Cardiovasc Interv 2011 4 505 13 10.1161/CIRCINTERVENTIONS.111.962183 22010189

[B52] Paré G Eikelboom JW Sibbing D Bernlochner I Kastrati A Testing should not be done in all patients treated with clopidogrel who are undergoing percutaneous coronary intervention Circ Cardiovasc Interv 2011 4 514 21 22010190 10.1161/CIRCINTERVENTIONS.111.962142

[B53] Holmes MV Perel P Shah T Hingorani AD Casas JP CYP2C19 genotype, clopidogrel metabolism, platelet function, and cardiovascular events: a systematic review and meta-analysis JAMA 2011 306 2704 14 10.1001/jama.2011.1880 22203539

[B54] Bhatt DL Paré G Eikelboom JW Simonsen KL Emison ES Fox KA Steg PG Montalescot G Bhakta N Hacke W Flather MD Mak KH Cacoub P Creager MA Berger PB Steinhubl SR Murugesan G Mehta SR Kottke-Marchant K Lincoff AM Topol EJ on behalf of the CHARISMA Investigators The relationship between CYP2C19 polymorphisms and ischaemic and bleeding outcomes in stable outpatients: the CHARISMA genetics study Eur Heart J 2012 33 2143 2215 10.1093/eurheartj/ehs059 22450429

[B55] Budnitz DS Pollock DA Weidenbach KN Mendelsohn AB Schroeder TJ Annest JL National surveillance of emergency department visits for outpatient adverse drug events JAMA 2006 296 1858 66 10.1001/jama.296.15.1858 17047216

[B56] Wysowski DK Nourjah P Swartz L Bleeding complications with warfarin use: a prevalent adverse effect resulting in regulatory action Arch Intern Med 2007 167 1414 9 10.1001/archinte.167.13.1414 17620536

[B57] Manolopoulos VG Ragia G Tavridou A Pharmacogenetics of coumarinic oral anticoagulants Pharmacogenomics 2010 11 493 6 10.2217/pgs.10.31 20350128

[B58] The International Warfarin Pharmacogenetics Consortium Estimation of the warfarin dose with clinical and pharmacogenetic data N Engl J Med 2009 360 753 64 19228618 10.1056/NEJMoa0809329 PMC2722908

[B59] Epstein RS Moyer TP Aubert RE O Kane DJ Xia F Verbrugge RR Gage BF Teagarden JR Warfarin genotyping reduces hospitalization rates results from the MM-WES (Medco-Mayo Warfarin Effectiveness study) J Am Coll Cardiol 2010 55 2804 12 10.1016/j.jacc.2010.03.009 20381283

[B60] Johnson JA Gong L Whirl-Carrillo M Gage BF Scott SA Stein CM Anderson JL Kimmel SE Lee MT Pirmohamed M Wadelius M Klein TE Altman RB Clinical Pharmacogenetics Implementation Consortium Guidelines for CYP2C9 and VKORC1 genotypes and warfarin dosing Clin Pharmacol Ther 2011 90 625 9 10.1038/clpt.2011.185 21900891 PMC3187550

